# Supplementary material for: Image polaritons in boron nitride for extreme polariton confinement with low losses
Source: Nat Commun. 2020 Jul 20;11:3649. doi: 10.1038/s41467-020-17424-w (PMC7371862; doi:10.1038/s41467-020-17424-w)
Supplement: Supplementary file 1 — Supplementary Information [file 41467_2020_17424_MOESM1_ESM.pdf]

Supplementary information:
Image polaritons in boron nitride for extreme polariton confinement with low losses

In-Ho Lee et al.

Supplementary Note 1. Image polariton dispersion

The magnetizing fields, $\mathbf{H}(x,y,z)$, in medium I $(y<0)$, II $(0<y<t)$, and III $(t<y<g)$ (illustrated in Supplementary Figure 1a) can be represented as

$$\mathbf{H}_l(x,y,z)=\begin{cases}\hat{\mathbf{e}}_z\left(H_\mathrm{i}e^{ik_{y,\mathrm{I}}y}+H_\mathrm{r}e^{-ik_{y,\mathrm{I}}y}\right)e^{ikx}, & l=\mathrm{I}\\ \hat{\mathbf{e}}_z\left(H_{2\mathrm{p}}e^{ik_{y,\mathrm{II}}y}+H_{2\mathrm{n}}e^{-ik_{y,\mathrm{II}}y}\right)e^{ikx}, & l=\mathrm{II}\\ \hat{\mathbf{e}}_z\left(H_{3\mathrm{p}}e^{ik_{y,\mathrm{III}}y}+H_{3\mathrm{n}}e^{-ik_{y,\mathrm{III}}y}\right)e^{ikx}, & l=\mathrm{III}\end{cases} \tag{1}$$

where k and $k_{y,l}$ are the wavenumber components in the x and y direction with the subscript $l\in\{\mathrm{I},\mathrm{II},\mathrm{III}\}$ representing the medium of interest. For the medium I and III, $k_{y,l}=\sqrt{\varepsilon_l {k_0}^2-k^2}$ while for the meidum II, $k_{y,\mathrm{II}}=\sqrt{\varepsilon_\parallel {k_0}^2-\frac{\varepsilon_\parallel}{\varepsilon_\perp}k^2}$with ε_l and k_0 being the complex permittivity of the medium and the free-space wavenumber, respectively. The electric fields are given as

$$\mathbf{E}_l(x,y,z)=\begin{cases}-\dfrac{1}{i\omega\varepsilon_\mathrm{I}}\left[\hat{\mathbf{e}}_y ikH_\mathrm{I}+\hat{\mathbf{e}}_x\left(ik_{y,\mathrm{II}}H_\mathrm{i}e^{ik_{y,\mathrm{II}}y}-ik_{y,\mathrm{I}}H_\mathrm{r}e^{-ik_{y,\mathrm{I}}y}\right)\right]e^{ikx}, & l=\mathrm{I}\\ -\dfrac{1}{i\omega\varepsilon_\parallel}\left[\hat{\mathbf{e}}_y ikH_\mathrm{II}+\hat{\mathbf{e}}_x\left(ik_{y,\mathrm{II}}H_{2\mathrm{p}}e^{ik_{y,\mathrm{II}}y}-ik_{y,\mathrm{II}}H_{2\mathrm{n}}e^{-ik_{y,\mathrm{II}}y}\right)\right]e^{ikx}, & l=\mathrm{II}\\ -\dfrac{1}{i\omega\varepsilon_\mathrm{III}}\left[\hat{\mathbf{e}}_y ikH_\mathrm{III}+\hat{\mathbf{e}}_x\left(ik_{y,\mathrm{III}}H_{3\mathrm{p}}e^{ik_{y,\mathrm{III}}y}-ik_{y,\mathrm{III}}H_{3\mathrm{n}}e^{-ik_{y,\mathrm{III}}y}\right)\right]e^{ikx}. & l=\mathrm{III}\end{cases} \tag{2}$$

From the continuities of the tangential components of the fields at the three interfaces $(y=0,t,$ and $g)$, we have

$$\begin{cases}H_\mathrm{i}+H_\mathrm{r}=H_{2\mathrm{p}}+H_{2\mathrm{n}},\\ H_\mathrm{i}-H_\mathrm{r}=\alpha_1\left(H_{2\mathrm{p}}+H_{2\mathrm{n}}\right), \qquad y=0,\end{cases} \tag{3}$$

$$\begin{cases}H_{2\mathrm{p}}e^{ik_{y,\mathrm{II}}t}+H_{2\mathrm{n}}e^{-ik_{y,\mathrm{II}}t}=H_{3\mathrm{p}}e^{ik_{y,\mathrm{III}}t}+H_{3\mathrm{n}}e^{-ik_{y,\mathrm{III}}t},\\ H_{2\mathrm{p}}e^{ik_{y,\mathrm{II}}t}-H_{2\mathrm{n}}e^{-ik_{y,\mathrm{II}}t}=\alpha_2\left(H_{3\mathrm{p}}e^{ik_{y,\mathrm{III}}t}+H_{3\mathrm{n}}e^{-ik_{y,\mathrm{III}}t}\right), \qquad y=t,\end{cases} \tag{4}$$

$$H_{3\mathrm{p}}e^{ik_{y,\mathrm{III}}g}=H_{3\mathrm{n}}e^{-ik_{y,\mathrm{III}}g}. \qquad y=g, \tag{5}$$

From the boundary conditions, the Fresnel reflection coefficient is given as

$$r_k=\frac{H_\mathrm{r}}{H_\mathrm{i}}=\frac{\left[(1-\alpha_1)e^{-ik_{y,\mathrm{II}}t}M+(1+\alpha_1)e^{ik_{y,\mathrm{II}}t}N\right]}{\left[(1+\alpha_1)e^{-ik_{y,\mathrm{II}}t}M+(1-\alpha_1)e^{ik_{y,\mathrm{II}}t}N\right]}. \tag{6}$$

where

$$M=(1-\alpha_2)e^{ik_{y,\mathrm{II}}(g-t)}+(1+\alpha_2). \tag{7a}$$

$$N=(1+\alpha_2)e^{ik_{y,\mathrm{II}}(g-t)}+(1-\alpha_2). \tag{7b}$$

The dispersion of the image polariton is obtained when the denominator vanishes as follows:

$$(1+\alpha_1)e^{-ik_{y,\mathrm{II}}t}M+(1-\alpha_1)e^{ik_{y,\mathrm{II}}t}N=0. \tag{8}$$

Supplementary Figure 1b shows the imaginary parts of r_k as a function of momentum and frequency. Similar to the case without a conducting plate, the dispersion consists of distinct branches that originate from the quantization of the out-of-plane wavevector. The spatial distributions of the x components of the electric fields for the first (Supplementary Figure 1c) and second (Supplementary Figure 1d) branch show the symmetric and anti-symmetric nature of the two modes, respectively.

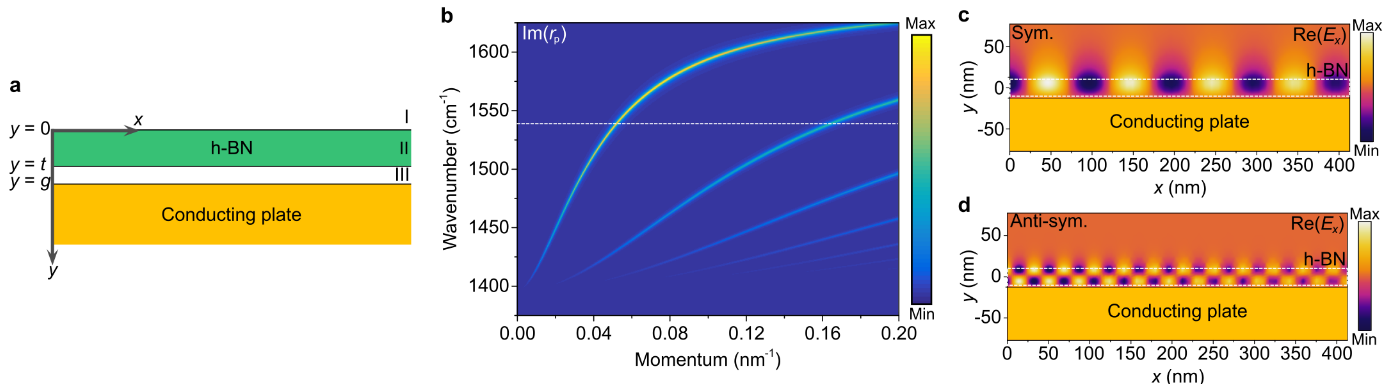

Supplementary Figure 1. Dispersion and mode symmetry. a, A schematic illustration of the structure of interest. **b,** The imaginary parts of the Fresnel reflection coefficient r_k as a function of frequency and momentum. The spatial distributions of the x-components of the electric fields for the mode **c,** on the first branch and **d,** on the second branch.

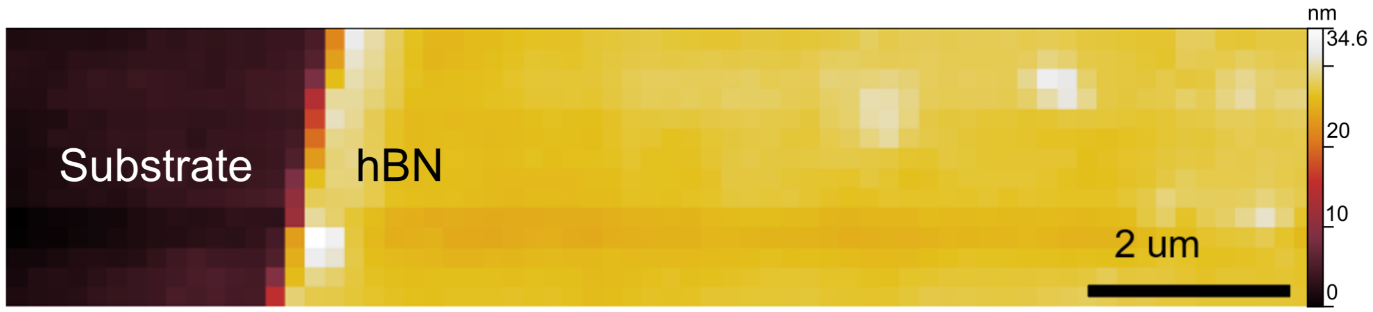

Supplementary Figure 2. Ultrasmooth topography of h^{10}BN. The topography of the h^{10}BN flake on a silicon substrate before transferring to the resonator taken by atomic force microscopy. The measured thickness and root-mean-square (RMS) roughness of the hBN flake are found to be 23 nm and 0.617 nm, respectively.

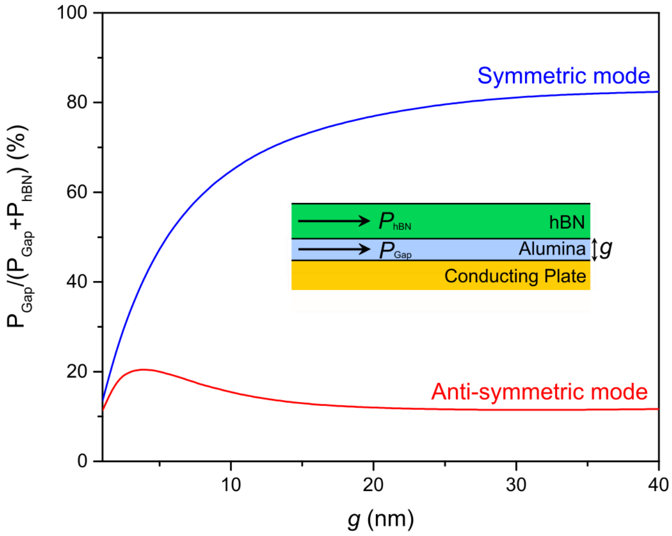

Supplementary Figure 3. Power distribution over the gap and $h^{10}BN$. The numerical results for the power distribution of the symmetric and anti-symmetric mode over the gap and hBN region as a function of the gap size. The symmetric mode has more power inside the gap compared to the anti-symmetric mode and the ratio of the power inside the gap region to the total power increases as the gap size increases. The power ratio of the anti-symmetric mode inside the gap is less sensitive to the gap size. The local maximum around $g = 4$ nm for the anti-symmetric case is attributed to the fact that the dipole interactions between charges at the bottom of hBN and their images in the conducting plate comparable to those between opposite charges within hBN around $g = 4$ nm. The inset shows the geometry used for numerical simulations, where P_{hBN} and P_{Gap} represent the magnitudes of time-averaged pointing vectors in the hBN and gap region, respectively.

Supplementary Note 2. Analytical model for resonator loss

Compared to a conventional Fabry-Perot resonator[1] wherein light is mostly reflected at the ends of a resonator unit, the polariton is mostly transmitted from one resonator unit to its neighbors at the ends of a resonator unit in our case[2]. Thus, the polaritons transmitted from a neighboring resonator unit constructively interfere with the polariton coupled from incident light to the resonator unit at the destination. In this case, the ratio of the resonantly enhanced field E_{res} to the initial field without interference E_0, is given as

$$\frac{E_{\mathrm{res}}}{E_0} = \frac{1}{1 - t_{21}t_{12}\exp\left[i(k_2 w + k_1 s)\right]}, \tag{9}$$

where t_{12} and t_{21} are modal transmission coefficients from the metal-free region to the metal-coupled region and vice versa, respectively. k_2 and k_1 are the momenta for the image and normal polariton supported by the metal-coupled and metal-free region, respectively. The square of the magnitude of Eq. (6) gives an internal resonance enhancement factor when the resonance condition given as $k_2 w + k_1 s = 2m\pi$ is met with m being the order of resonance. The total damping rate γ_{total} including the scatterings at the edges of a resonator unit e and the propagation loss γ_{p} can be modelled from the relation of

$$\gamma_{\mathrm{total}} = \gamma_{\mathrm{e}} + \gamma_{\mathrm{p}} \approx \frac{-\ln\left(T_{12}T_{21}\right)}{\tau_{\mathrm{p}}} + 2v_{\mathrm{g}}|\mathrm{Im}(k_2)|, \tag{10}$$

where T_{12} and T_{21} are modal transmittances given as $T_{12} = {t_{12}}^2$ and $T_{21} = {t_{21}}^2$. v_{g} is the group velocity of the polariton and τ_{p} is the dwelling time of the polariton within a resonator unit defined as $\frac{p}{v_{\mathrm{g}}}$ with p being the periodicity of the metal ribbon array. Due to the finite thickness of hBN, the polariton also undergoes scattering at the surface of hBN, which is expected to become intense for small thicknesses (t) and large hyperbolic angles (θ) with respect to the in-plane direction. Also, the smaller distance between hBN and the metal ribbon array increases the intensities of hyperbolic rays, which leads to more intense surface scattering. These observations lead to the addition of the surface scattering component γ_{s} to the total scattering rate as follows:

$$\gamma_{\mathrm{total}} = \gamma_{\mathrm{e}} + \gamma_{\mathrm{p}} + \gamma_{\mathrm{s}} \approx \frac{-\ln\left(T_{12}T_{21}\right)}{\tau_{\mathrm{p}}} + 2v_{\mathrm{g}}|\mathrm{Im}(k_2)| + \frac{v_{\mathrm{g}}|\tan\theta|}{t}\frac{A}{1+\frac{g}{g_{\mathrm{c}}}}. \tag{11}$$

Supplementary Note 3. Purcell factor estimation

To calculate the Purcell factor, the mode volume should first be determined. Since the optical near-fields of image polaritons are indefinitely continuous along the long axes of ribbons in our case, however, the mode volume of image polaritons is in principle also indefinite. Instead, we approximately estimate the mode volume V_{eff} with V_{eff}=(periodicity)^2(gap+hBN thickness+evanescent field decay length) assuming that an image polariton resonator with square patches would have similar polariton properties. Then, the Purcell factor F_{P} can be calculated from

$$F_{\mathrm{P}} = \frac{3}{4\pi^2}\left(\frac{\lambda}{n}\right)^3\left(\frac{Q}{V_{\mathrm{eff}}}\right). \tag{12}$$

where λ_0, n, and Q represent a free-space wavelength of incident light, a refractive index of a cavity, and a quality factor of a resonance.

The Purcell factor estimated in the main article was calculated from one of the anti-symmetric resonances with the largest effective index of 132 and Q of 461 at the wavenumber of 1507 cm^{-1} for which the periodicity of the metal ribbon array is 100 nm and the gap size is 3 nm. Thus, the mode volume is (100 nm)^2(3 nm+23 nm+7 nm), with the estimated decay length of the evanescent fields into the air being 7 nm. For the refractive index of a cavity n, the refractive index of alumina at 1507 cm^{-1} is used as an approximation.

Supplementary References

1. Ismail, N., C., K. C., Geskus, D. & Pollnau, M. Fabry-perot resonator: spectral line shapes, generic and related airy distributions, linewidths, finesses, and performance at low or frequency-dependent reflectivity. *Opt. Express* **24**, 16366–16389 (2016).
2. Lee, I.-H., Yoo, D., Avouris, P., Low, T. & Oh, S.-H. Graphene acoustic plasmon resonator for ultrasensitive infrared spectroscopy. *Nat. Nanotechnol.* **14**, 313–319 (2019).
3. Marquardt, D. W. An algorithm for least-squares estimation of nonlinear parameters. *SIAM J. on Appl. Math.* **11**, 431–441 (1963).

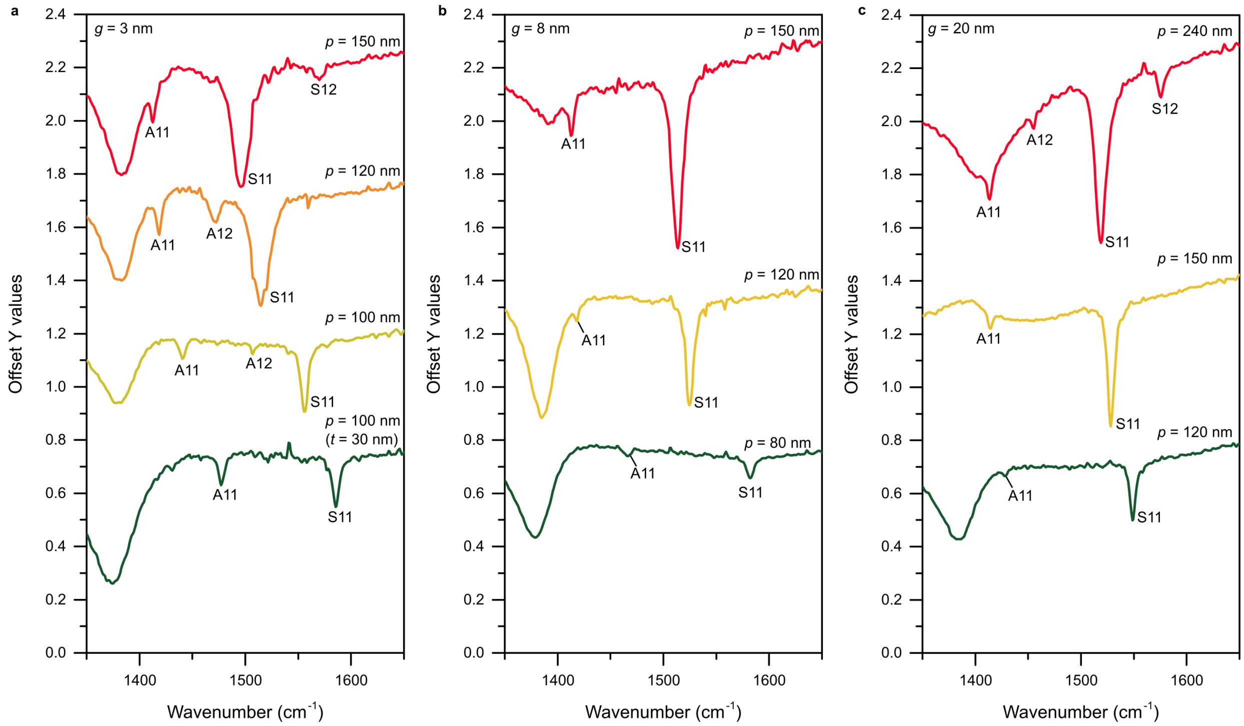

Supplementary Figure 4. Measured spectra for h^{10}BN. The spectra measured for **a,** g = 3 nm with the periodicities (p) of 100, 120, and 150 nm, **b,** g = 8 nm with p of 100, 120, and 150 nm, and **c,** g = 20 nm with p of 120, 150, and 240 nm.

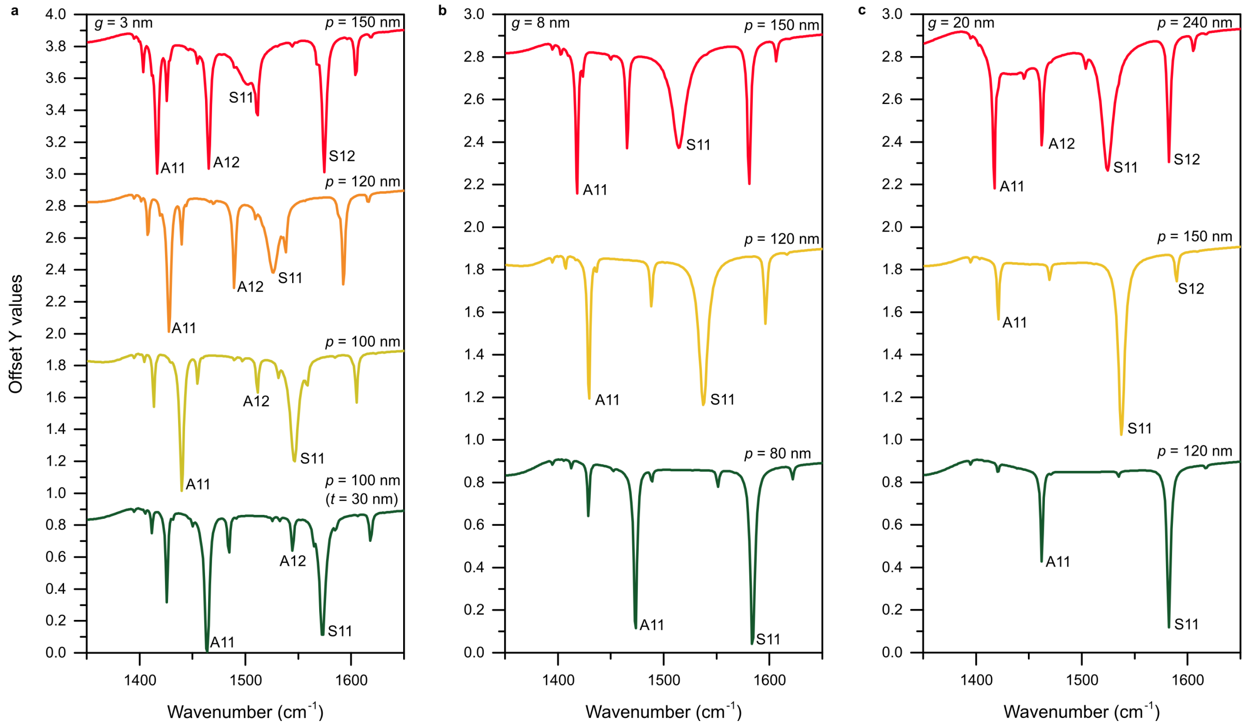

Supplementary Figure 5. Simulated spectra for h^{10}BN. The spectra calculated for **a,** g = 3 nm with the periodicities (p) of 100, 120, and 150 nm, **b,** g = 8 nm with p of 100, 120, and 150 nm, and **c,** g = 20 nm with p of 120, 150, and 240 nm.

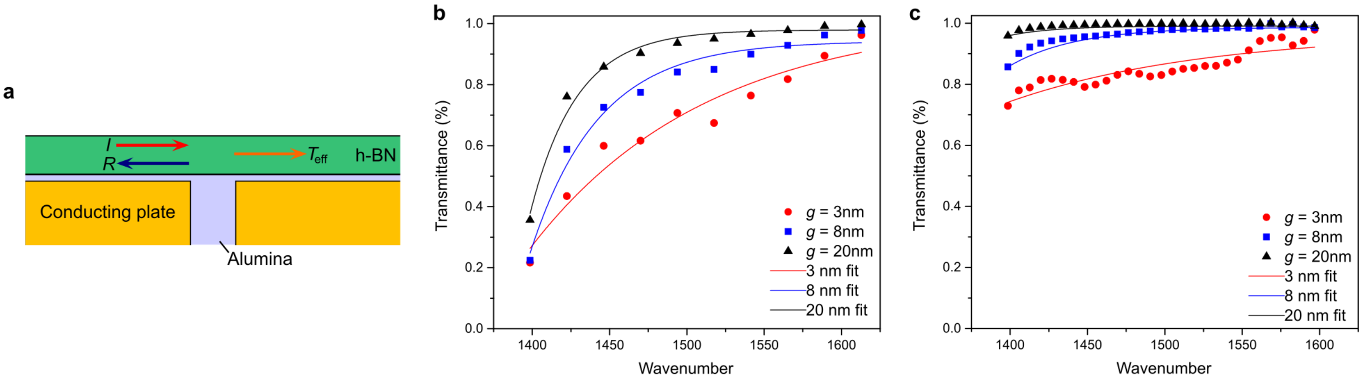

Supplementary Figure 6. Modal transmittances. a, A schematic illustration of the geometry considered to calculate the modal transmittance of the polariton at the interface between two conducting plates. The modal transmittances as a function of a frequency for **b,** the symmetric mode and **c,** the anti-symmetric mode for different gap sizes of 3, 8, and 20 nm.

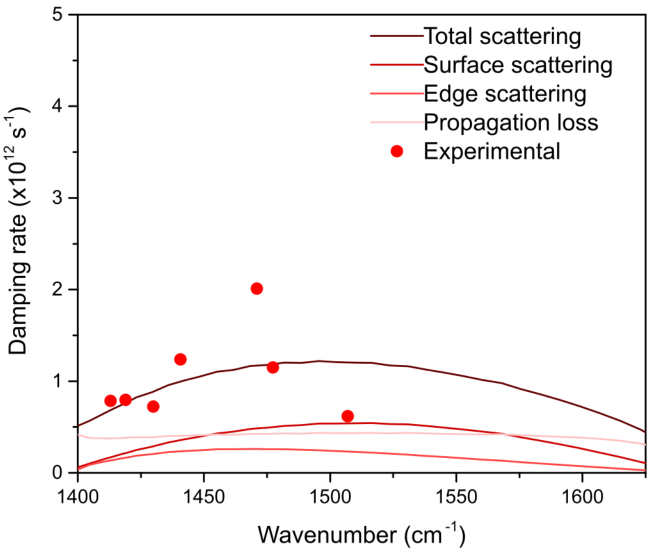

Supplementary Figure 7. Damping channels for anti-symmetric mode. The loss contributions from the scatterings at the edges of resonator units, the propagation loss, and the surface scatterings calculated from the analytical model (solid lines) together with the experimental results (red circles) for $g = 3$ nm. Compared to the symmetric case, the edge and surface scattering rates are much lower due to the tighter confinement of the anti-symmetric mode in a hBN slab.

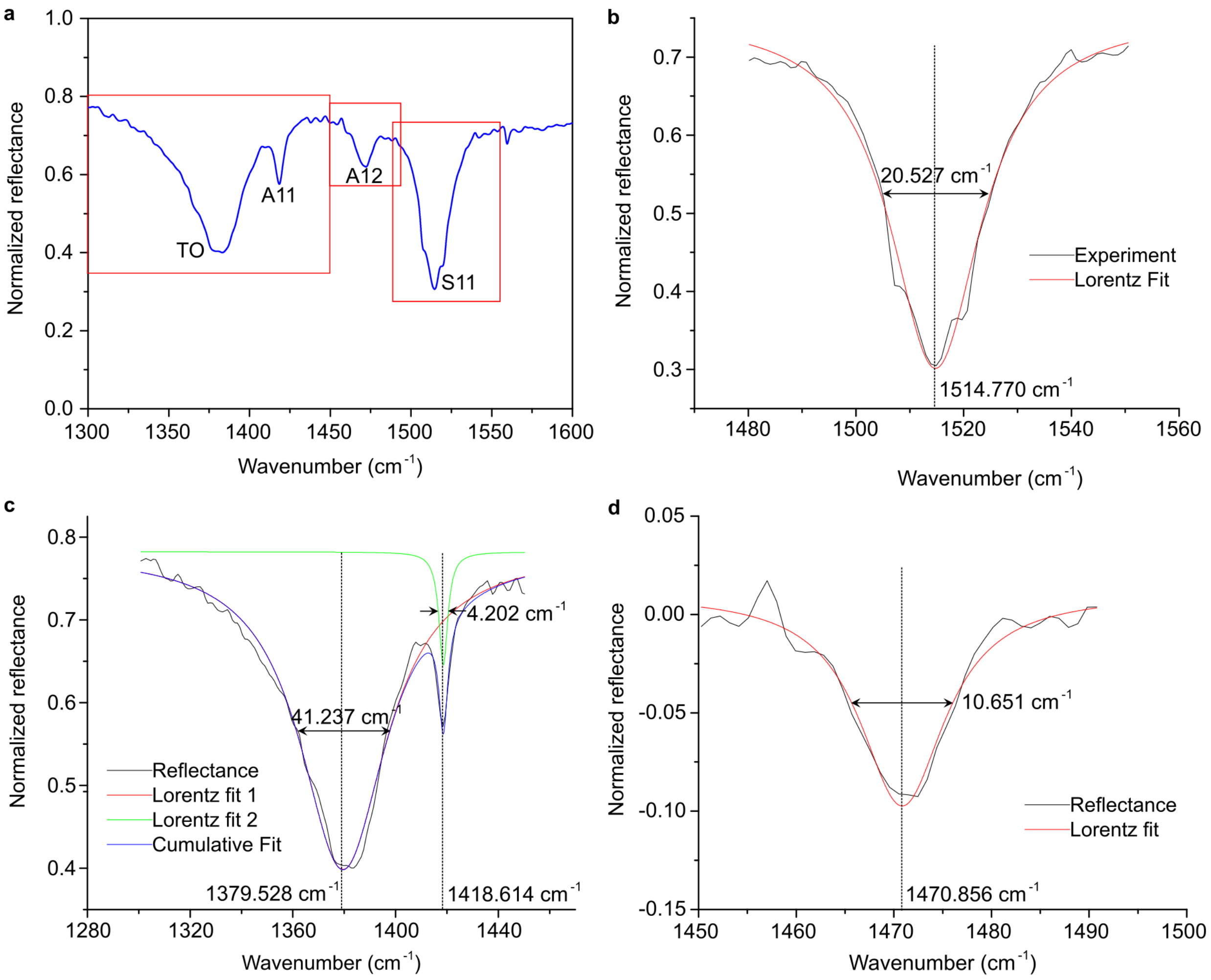

Supplementary Figure 8. Quality factor extractions. a, The measured reflection spectrum for the resonator with g = 3 nm and p = 120 nm. Three resonances are observed, which corresponds to the first and second order anti-symmetric resonance (A11 and A12), and the first order symmetric resonance (S11). The broad peak at 1370 cm^{-1} arises from coupling between the transverse optical phonon and the cavity mode in the optical spacer, which is denoted as 'TO' for convenience. b, The Lorentzian fitting of **b,** S11 **c,** A11, and **d,** A12. In **a**, the resonance is first isolated from the spectrum and then fitted with a Lorentzian function using the Levenberg-Marquardt method[3] implemented in the commercial software (Origin; OriginLab Co.). In **c** where A11 is spectrally overlapped with the TO resonance, the overlapped spectra region is fitted with two Lorentzian curves using the same method. When the resonance is on a linear tilted slope baseline as in **d**, the isolated spectrum is fitted with a Lorentzian curve after baseline correction.

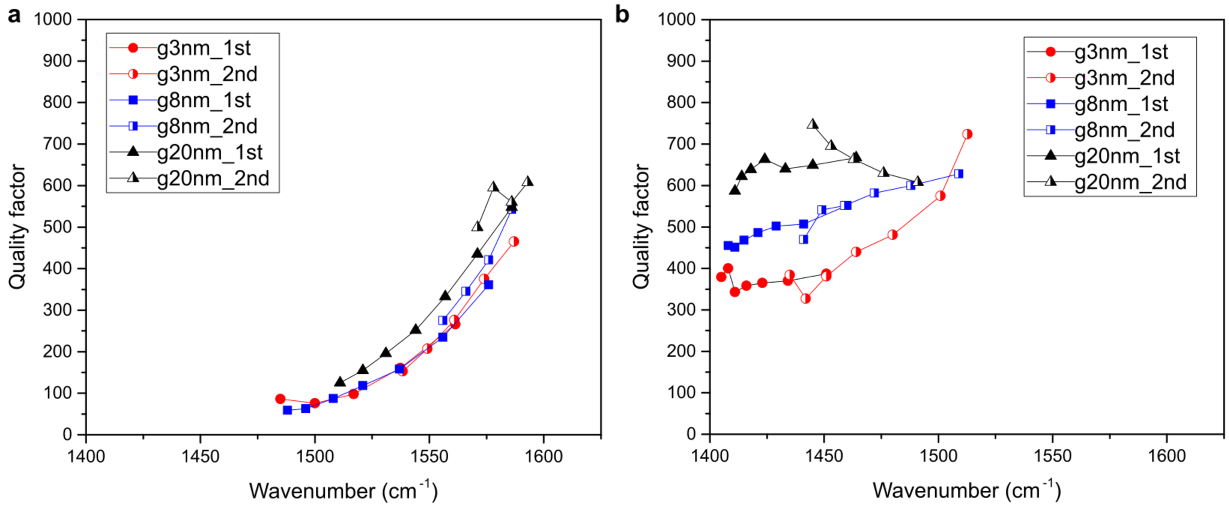

Supplementary Figure 9. Numerically calculated quality factors. The quality factors extracted from numerically calculated reflection spectra for **a,** the symmetric modes and **b,** the anti-symmetric mode for different gap sizes of 3, 8, and 20 nm. The solid and half-filled symbols represent the 1st and 2nd order resonances, respectively. In general, the quality factors for the 1st and 2nd resonances are of similar magnitudes.
